# Analysis of Lead Ions in a Waste Solution Using Infrared Photo-Diode Electrode

**DOI:** 10.5487/TR.2008.24.3.227

**Published:** 2008-09-01

**Authors:** Suw Young Ly, Hyun Kuy Lee, Kyu Ju Kwak, Jun Seok Ko, Jeong Jae Lee, Jin Hee Cho, Ki Hong Kim, Min Seok Kim, So Jung Lee

**Affiliations:** 111 Biosensor Research Institute in Seoul National University of Technology, Seoul, 139-743 Korea; 211 Department of Chemistry, Yonsei University, Seoul, 120-749 Korea; 311 Advanced Scientific Experiment Group in DaeJin High School, Korea; 411 The Science Group in CheongWon Women’s High School, Korea

**Keywords:** Infrared photo diode electrode, Voltammetry, Lead

## Abstract

To detect lead ions using electrochemical voltammetric analysis, Infrared Photo-Diode Electrode (IPDE) was applied via cyclic and square wave stripping voltammetry. Lead ions were deposited at 0.5 V (versus Ag/AgCl) accumulation potential. Instrumental measurements systems were made based on a simple and compact detection system. The stripping voltammetric and cyclic voltammetric optimal parameters were searched. The results yielded a cyclic range of 40∼240 mgl^−1^ Pb(II) and a square wave stripping working range of 0.5∼5.00 mgl^−1^ Pb(II). The relative standard deviation at 2 and 4 mgl^−1^ Pb(II) was 0.04% and 0.02% (n = 15), respectively, using the stripping voltammetric conditions. The detection limit was found to be 0.05 mgl^−1^ with a 40 sec preconcentration time. Analytical interference ions were also evaluated. The proposed method was applied to determine lead ions in various samples.

## INTRODUCTION

Lead is one of the most commonly used elements in various industries. It is present in batteries, gasoline, paint pigments, glass, electrical cable, and other products. The extensive application of lead in everyday life, however, has caused serious health problems for many people (Jing *et al.*, 2001; Kwang *et al.*, 1998). For instance, lead ion toxicosis affects the liver, the central nervous system, eyes, and kidneys. Lead ion salt poisoning affects the blood and is usually transmitted through contaminated food and drinking water. Consequently, numerous techniques for the determination of lead ions have been developed in the field of analytical science (Maria *et al.*, 2004; Emanuele *et al.*, 2003; Scarponi *et al.*, 1997). In recent years, various instrumental techniques, such as HPLC, mass spectrophotometry, ICP, and other systems (Chau *et al.*, 1997; Lauralynn *et al.*, 2001; Kozelka *et al.*, 1997; Eric *et al.*, 1997; Robert *et al.*, 1999; Scarponi *et al.*, 1997; Kevin *et al.*, 1998) have been developed. These modern techniques are widely used and have widespread applications for the detection of lead ions in various materials, such as blood, paint, food. These techniques are also used in various fields, such as bioassay. Most of these techniques, however, are complicated, time-consuming, and expensive, and require multiple components such as sample treatment systems, separation systems, temperature-controlling equipment, spectrophotometric or electrical detection systems, and other data treatment systems. Thus, simpler and more inexpensive analytical techniques are required. Recent research studies have discovered electrochemical cyclic, stripping, and chrono systems, which can serve as simple, compact, fast, and inexpensive alternative techniques (Suw, 2008; Joseph *et al.*, 2001) for trace metal detection and organic bioassay. However, as these systems depend on various types of working electrodes, conventional and specific electrodes have been investigated and developed in this study, such as mercury and its composition sensing electrode (Suw *et al.*, 2002; El-Hefnawey *et al.*, 2004; Cristina *et al.*, 2001), graphite carbon materials (Ouassim *et al*., 2004; Yuwu *et al*., 2003), micro fiber type (Jose *et al*., 2001; Xueji *et al*., 1999; Greg and Alexander, 2001), modified paste (Huaisheng *et al*., 1998; Jahan *et al*., 2001; Priya *et al*., 2001), graphite pencil (Suw *et al*., 2004; Prinya *et al*., 2002), and other specialized electrodes. Thus, although various working electrode types are currently being used in lead ion detection (Jyh *et al*., 1999; Khiena *et al*., 2001; Yu *et al*., 2001; Cidalia *et al*., 2001), most of these are still expensive because they are developed by specialized producers. Therefore, more sensitive, inexpensive, and simpler working electrode types than existing ones remain in demand. In this study, the application of infrared photo diode electrode (IPDE) in voltammetry analysis, under optimal conditions, was explored. Furthermore, the IPDE surface material is a photo-sensing and gas sensing (Gagliardi and Gianfrani, 2002; Georges, 2001; Schlosser *et al*., 2002; Yamamoto and Yoshida, 2002; Eninari *et al*., 2003) electrochemical type. Moreover, various papers have investigated its cyclic voltammetric effects (Christian *et al*., 2003; Sungouk *et al*., 2004; Kai *et al*., 2004), with its CV (P or n doping) copolymer structure on a metal surface with strong electron-donating properties (Mohammed *et al*., 2002). In addition, its kinetic effect, analytical measurements, and absorption experiments have also been searched in other papers (Hai *et al*., 2000; Jeong *et al*., 2001). The suggested IPDE method makes use of a compact, simple, and inexpensive electrode. It is also valuable for the trace detection of low amounts of lead and for other analytical cyclic reduction or electronic oxidation detections in various fields.

## MATERIALS AND METHODS

**Apparatus, reagent, and electrode preparation.** Voltammetric experiments were performed using a compact and inexpensive EPA1000 portable electrochemical analyzer (South Korea Pimacs Co., Ltd). A conventional three-electrode system was used to monitor the cyclic and square wave stripping voltmeter signals. Various common working electrode types were prepared with a 7 μm-diameter carbon fiber microelectrode, carbon paste composite electrode, glassy carbon electrode, platinum metal electrode, and a new type of electric circuit-infrared photo diode electrode. The Ag/AgCl electrode (saturated KCl) was used as the reference electrode, whereas a platinum wire served as the auxiliary electrode. All solutions were prepared using double-distilled water (~18 M Ohm. $Cm^{-1}$). Other solutions were prepared by diluting the corresponding standard stock atomic absorption solution of 1 $gl^{-1}$. On the other hand, a 0.1 M acetic buffer solution (pH 6.0) served as the supporting electrolyte. The IPDE working electrode was prepared using the electric circuit of a sensing device. The specific properties of this sensing device include a 430~1050 nm infrared photo-sensing tip, a peak sensitivity wavelength of 950 nm, and a packing view size of 4.8~5.5 mm (Lung and Yin, 2004). This device was manufactured by a UK-based semiconductor company, which also supplies SPI5342-H products, TV VCR audio remote controllers, and equipment circuits. Both (N-P type) electrodes were tested forward and backward for anode or cathode reactions (Dong *et al*., 2003; Christian *et al*., 2003; Mohammed *et al*., 2002), in which identical current peaks were obtained. The colorless transparency cover glass was first removed, and then the other base plate was set aside. Next, the two bare-type electrodes 1 mm in diameter and 1 cm in length were washed with double-distilled water. On the other hand, the conventional paste electrode was prepared by mixing 70% graphite powder with 20% mineral oil. This mixture was homogenized in a mortar for 30 min. The mixed paste was inserted into a plastic syringe needle with a diameter of 3 mm, whereas a copper wire was connected to the electrical system (Huaisheng *et al*., 1998; Jahan *et al*., 1998; Priya *et al*., 2001). Then, glassy carbon and platinum electrodes were combined with a conventional electrode (Ouassim *et al*., 2004; Yuwu *et al*., 2003). The hand-made carbon fiber microelectrode was constructed from a carbon fiber with a diameter of 7 μm and a length of about 15 mm. This was attached to the lead wire using silver paint. The carbon fiber microelectrode was then inserted into a polyethylene tube with a diameter of 0.3 mm, which was then sealed by heating. The electrodes were cleaned in a three-step process: first in acetone by sonication for 10 minutes, then in nitric acid (1 : 1), and finally, in double distilled water (Jose *et al*., 2001; Xueji *et al*., 1999; Greg *et al*., 2001). The three-electrode system was immersed in a 15 ml cell containing electrolyte solutions of 0.1 M acetic and sodium acetate solution (pH 6.0). The accumulation potential was performed under normal conditions at 0.5 V for 50 sec, whereas the other parameters were maintained at optimal conditions. All experiments were performed at 24 ± 0.5 room temperature without removing oxygen.

## RESULTS AND DISCUSSION

**Voltammetric effects.** The cyclic voltammogram was used to determine the electrode behavior of lead ions in 0.1-M acetate buffer solutions with pH 6.0. The IPDE electrode was applied at a potential scan range of from -0.02 to -1.0 V. Voltammetric signals were obtained in the recorded blank range and in 50 $mgl^{-1}$ Pb(II) spiking. The solution was then tested at various scan rates of 200, 300, 350, 400, 450, 500, 600, 700, 800, 900 and 1000 $mVs^{-1}$ (A). At this point, the range of 200~350 $mVs^{-1}$ quickly increased, then, from 350 to 1000 $mV^{-1}$, it linearly increased. The linear equation, $y = 0.0388x + 1.02$ ($R^2 = 0.9756$) was generated. Therefore, concentration increments were examined at optimal conditions within 350 $mVs^{-1}$. Fig. 1(B) shows the 10-ml electrolyte blank solutions, as no noise and other signals could be obtained. Afterwards, 0, 40, 60, 80, 100, 120, 140, 160, 180, 200, 220, and 240 $mgl^{-1}$ Pb(II) was added to the electrolyte solution, which was spiked at a scan rate of 350 $mVs^{-1}$. As shown on the forward positive scan, the CV continually exhibited a sharp peak current, which increased at -0.5 V, corresponding to the oxidation peak currents of the lead ions. The increasing peak currents, however, were not observed in the backward scan. After the peak signals were plotted and regression analysis was performed, the following equation was obtained:

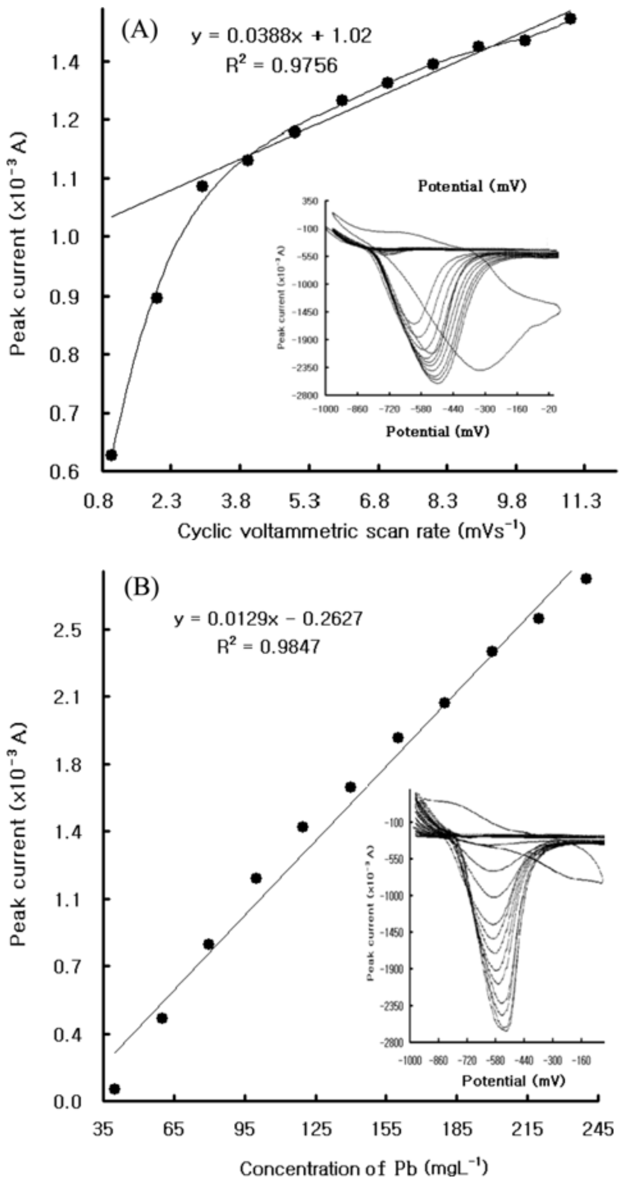

**Fig. 1.** (A): Cyclic voltammetric scan rate at 200, 300, 350, 400, 450, 500, 600, 700, 800, 900, 1000 $mVs^{-1}$; (B) Cyclic voltammetric concentration effects of IPDE in 0.1 M acetic acid solution with pH 6.0, with the addition of 0, 40, 60, 80, 100, 120, 140, 160, 180, 200, 220, and 240 $mgl^{-1}$ Pb(II), at a scan rate of 100 $mVs^{-1}$ and a scan range of 0.02 to -1.0.

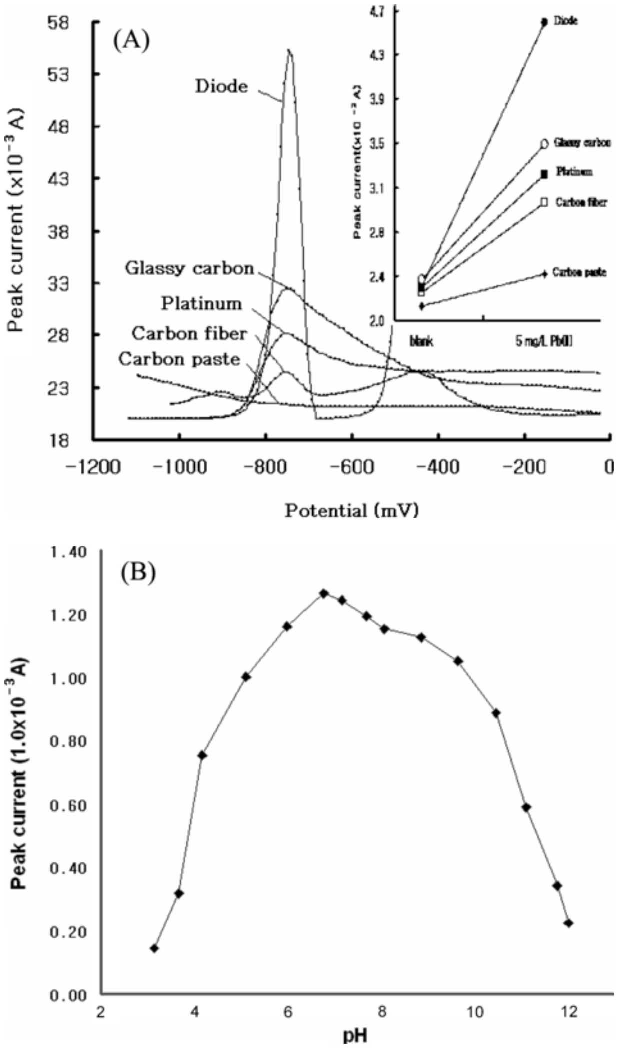

**Fig. 2.** (A): Comparison of the response to IPDE versus various electrodes, including glassy carbon, platinum, carbon fiber, and carbon paste electrode, at 5.0 $mgl^{-1}$ Pb(II); accumulation time of 50 sec. (B): Various pH effects of 3.1, 3.7, 4.1, 5.1, 6.0, 6.8, 7.2, 7.7, 8.1, 8.9, 9.7, 10.5, 11.0, 11.8, and 12.0, at 0.1 M acetic acid solution. Other conditions are the same as in Fig. 3.

y = 0.0129X - 0.2627 ($R^2$ = 0.9847). Subsequently, the effect of the acetic acid electrolyte concentration was studied within the range of 0.01~0.5 M at the fixed lead ions. In particular, key factors and various diluted activity components were tested, such as the peak height current acquired for the 0.1-M acetic acid concentration. In addition, electrolyte solutions with other acids and bases were tested, including sulfuric, hydrochloric, phosphoric acid, and other base solutions. The test, however, did not yield good results. Finally, optimal conditions were set up for the different stripping experimental parameters. In the next section, the use of square wave voltammetric study will be discussed.

**Square wave stripping voltammetric effects.** Fig. 2(A) shows the comparison of the sensitivity of the square wave stripping effect of IPDE and the different conventional electrodes in fixed concentrations of 5.0 $mgl^{-1}$ Pb(II) spike, under optimal conditions. As shown in the figure, the IPDE, conventional glassy carbon, platinum, micro carbon fiber, and carbon paste electrodes were all prepared under the same conditions and identical parameters were used. The peak signal ratio was obtained at the 4.6 > 3.5 > 3.3 > 3.0 > 2.4 mA. As shown in the figure, the IPDE generated peak signals at an increasing rate, and exhibited a sharper peak width, compared to the other common electrode types. In addition, the high and low concentrations used in stripping and cyclic voltammetry also yielded the same results. Next, the electrolyte hydrogen ionic activity and strength were tested using 0.1-M hydrochloric acid and 0.1-M sodium hydroxide solutions. In this case, the 0.1 M acetic acid buffer solution on the preconcentration of lead ions was altered within the range of 3.1, 3.7, 4.1, 5.1, 6.0, 6.8, 7.2, 7.7, 8.1, 8.9, 9.7, 10.5, 11.0, 11.8, and 12.0 pH. The other parameters were fixed under the optimal conditions set up in Fig. 3. As shown in Fig. 2(B), the peak current increased rapidly as the pH level

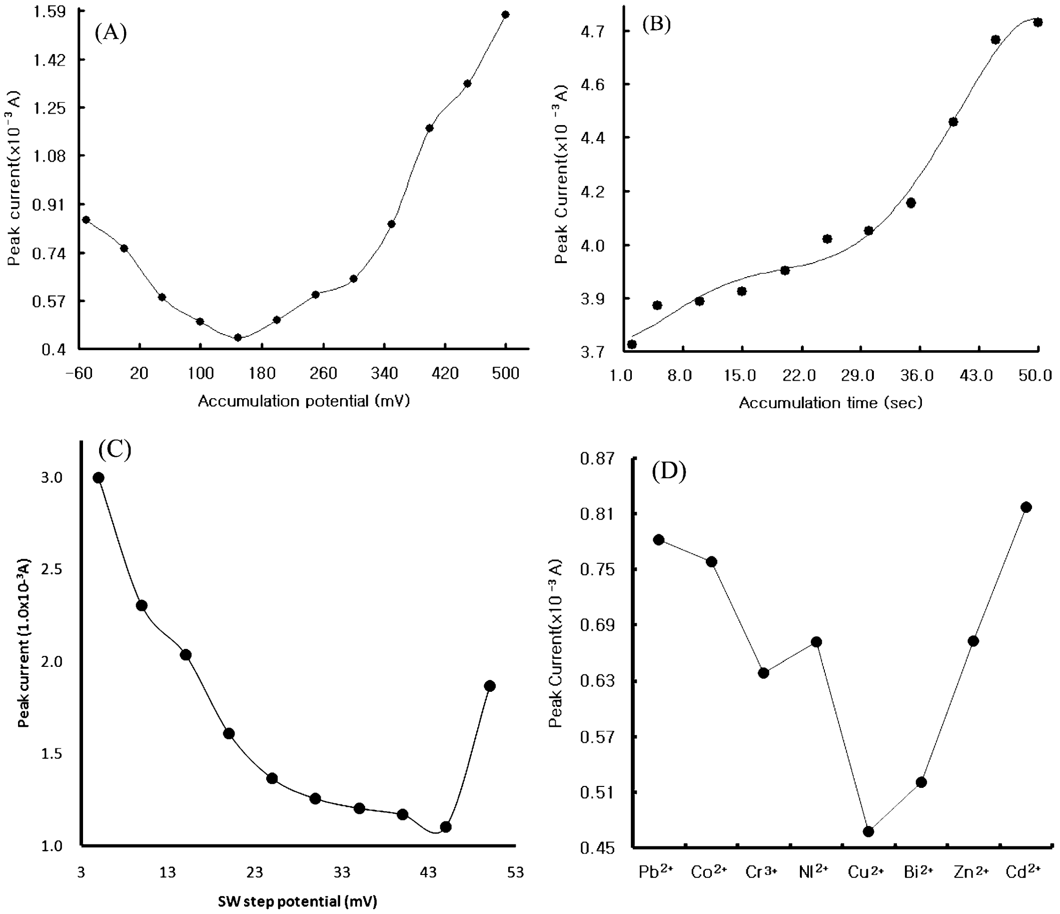

**Fig. 3.** (A): Response to various accumulation potentials at -50, 0, 50, 100, 150, 200, 250, 300, 350, 400, 450, and 500 mV. (B): Accumulation times at 2, 5, 10, 15, 20, 25, 30, 35, 40, 45, and 50 sec. (C): Square wave step potential at 5, 10, 15, 20, 25, 30, 35, 40, 45, and 50 mV, in IPDE at 5.0 $mgl^{-1}$ Pb(II). (D): Interference test for three-fold metal ions. Other conditions are the same as in Fig. 2.

was raised to 6.0. The peak current remained constant within the pH range of 6.0~10.5. Beyond this range, however, the pH sensitivity quickly decreased and disappeared. Therefore, a pH of 6.8 was established as the optimal level.

Fig. 3(A) shows the accumulation potential response within the range of -50, 0, 50, 100, 150, 200, 250, 300, 350, 400, 450, and 500 mV, at 5.0 $mgl^{-1}$ Pb(II) spike. As shown in the figure, the currents increased steadily and quickly from 150 to 500 mV; thereafter, the peak gradually decreased and almost disappeared. Thus, 500 mV was fixed as the optimum accumulation potential. When the currents were examined under low and high concentrations of Pb(II), the same results showing sensitive and sharp peak widths were obtained. In Fig. 3(B), the dependence of the peak current on the accumulation times set at 2, 5, 10, 15, 20, 25, 30, 35, 40, 45, and 50 sec was demonstrated. As the accumulation time was increased, the peak current gradually increased, raising the total current to $1.02^{-3}$ A. In general, the accumulation of lead ions onto the electrode depends on the length of the accumulation time. In addition, it was discovered that adsorption equilibrium is attained only after 40 sec. In other studies that dealt with different high or low lead ion concentrations, the same results were obtained at very short adsorption times. Thus, the optimum accumulation time was fixed at 40 sec in the quantitative estimations of Pb(II). In Fig. 3(C), the accumulation step potential was examined within the range of 5, 10, 15, 20, 25, 30, 35, 40, 45, and 50 mV. Other parameters were maintained at optimum conditions. The results indicate that the parameters were strongly influenced from 5~40 mV. Thereafter, the peak response quickly decreased and the maximum ratio was reached at $1.13^{-3}$ A. When the concentration effects were investigated, almost the same results were obtained. Under optimal conditions, the analytical interference of other metal ions was tested for possible noise presence in lead ion determination. In the detection of 1-$mgl^{-1}$ Pb(II) with a 40-sec accumulation time, the addition of 1 $mgl^{-1}$ Co(II), Cr(III), Ni(II), Cu(II), Bi(II), Zn(II), and Cd(II) did not cause any interference. Moreover, at the three-fold interference level, Co(II) and Ni(II) did not show any significant influence. In Fig. 3(D), Cr(III), Cu(II), Bi(II), Zn(II), and Cd(II) showed significant interference at -15, -30, 11, 29 and 21%. These interfering ions can be eliminated by applying the standard addition method in the determination of lead ion concentration.

**Statistics and application.** Fig. 4(A) shows the effects of various working concentration ranges, which were examined under optimal conditions. Real voltammograms appeared at 0.5, 1, 1.5, 2, 2.5, 3, 3.5, 4, 4.5, and 5 $mgl^{-1}$ Pb (II) spike. The acetate buffer solutions were tested for low and high concentration effects. Within the low range of 0.1~1.0 $mgl^{-1}$ Pb(II), uncertain and inaccurate linear equations were obtained. Therefore, the values were ignored. In the linear statistical range of 0.5~5.0, however, useful levers were fixed at 10 points, yielding the following results: $y = 0.5885x + 0.2891$; correlation coefficient = 0.9947; y = peak current of $\times 10^{-3}$ ampere; and x = Pb(II) concentration of $mgl^{-1}$. All the stripping response results are highly reproducible. Measurements with 2~4 $mgl^{-1}$ Pb(II) were repli-

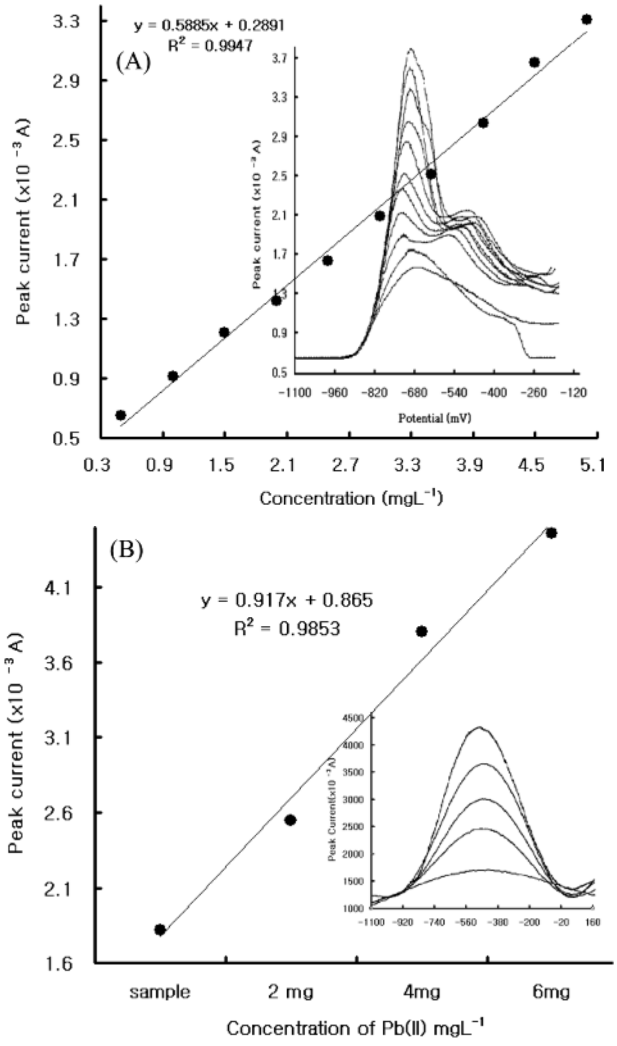

**Fig. 4.** Stripping voltammograms of Pb(II) at IPDE. Solutions of 0.1-M acetate buffer containing (A) 0.5, 1, 1.5, 2, 2.5, 3, 3.5, 4, 4.5, and 5 $mgl^{-1}$ Pb(II). Analytical application (B) of blank, real sample, and standard 2, 4, and 6 $mgl^{-1}$ Pb(II). Deposition for 40 sec at 0.5 V, square wave voltammetric stripping scan with an accumulation potential of 0.5 V, potential step of 5 mV, and amplitude of 30 $mVs^{-1}$.

cated 15th and calibrated at relative standard deviations of 0.04% and 0.02%, respectively. The detection limit of 0.04 $mgl^{-1}$ with a 40-sec preconcentration time can be estimated based on the signal-to-noise characteristics of these data (S/N = 3). Under optimal conditions, the proposed method in this study was applied to determine the amount of lead ions in pharmaceutical waste samples. Fig. 4(B) shows the applications of pharmaceutical factory waste raw voltammograms. The first scan was carried out in a blank solution. Then, a 1-ml sample solution was spiked, generating detectable small peak signals and suitable aliquots between 2 and 6 ppm of the fixed standard. The lead ion solution was spiked at four different cells containing 10 ml of the same electrolyte cell. Thereafter, the least square equations of the standard addition plot yielded a slope of 0.917 $mA/mgl^{-1}$ and a correlation coefficient of 0.9853. The average concentration of lead ions was calibrated at 1.32 $mgl^{-1}$. Other applications of city and school laboratory waste samples on known concentrations were calibrated. The results yielded a city waste concentration level of 1.130 $mgl^{-1}$ and a laboratory waste concentration level of 0.622 $mgl^{-1}$. A mean 95% confidence limit was detected by triple analysis.

## CONCLUSION

Using the method proposed in this study, it was demonstrated that the use of non-mercury electrodes with sensitive response characteristics, such as the IPDE, is suitable for the application of several metals or other biological assay in voltammetric systems. The results of this study show that it is possible to accurately measure the presence of lead ions in aqueous solutions using a low detection limit of 0.05 $mgl^{-1}$ with a 40 sec preconcentration time under optimal conditions. The newly developed method was compared with conventional electrode types in terms of response sensitivity. The results confirm that the proposed method is superior to the electrode types that are currently in use. The IPDE method is suggested as a practical alternative technique for any cyclic and voltammetric instrumental working systems, as it is useful, stable, simple, inexpensive, and can be reproduced. The IPDE method can be useful in a water solution with pH value ranging from pH 4 to pH 11.
